# Comparative Analysis for the Performance of Long-Read-Based Structural Variation Detection Pipelines in Tandem Repeat Regions

**DOI:** 10.3389/fphar.2021.658072

**Published:** 2021-06-07

**Authors:** Mingkun Guo, Shihai Li, Yifan Zhou, Menglong Li, Zhining Wen

**Affiliations:** ^1^ College of Chemistry, Sichuan University, Chengdu, China; ^2^ Medical Big Data Center, Sichuan University, Chengdu, China

**Keywords:** structural variation, tandem repeats, detection pipelines evaluation, rare diseases, long-read sequencing

## Abstract

There has been growing recognition of the vital links between structural variations (SVs) and diverse diseases. Research suggests that, with much longer DNA fragments and abundant contextual information, long-read technologies have advantages in SV detection even in complex repetitive regions. So far, several pipelines for calling SVs from long-read sequencing data have been proposed and used in human genome research. However, the performance of these pipelines is still lack of deep exploration and adequate comparison. In this study, we comprehensively evaluated the performance of three commonly used long-read SV detection pipelines, namely PBSV, Sniffles and PBHoney, especially the performance on detecting the SVs in tandem repeat regions (TRRs). Evaluated by using a robust benchmark for germline SV detection as the gold standard, we thoroughly estimated the precision, recall and F1 score of insertions and deletions detected by the pipelines. Our results revealed that all these pipelines clearly exhibited better performance outside TRRs than that in TRRs. The F1 scores of Sniffles in and outside TRRs were 0.60 and 0.76, respectively. The performance of PBSV was similar to that of Sniffles, and was generally higher than that of PBHoney. In conclusion, our findings can be benefit for choosing the appropriate pipelines in real practice and are good complementary to the application of long-read sequencing technologies in the research of rare diseases.

## Introduction

Previous studies typically defined structural variations as genomic changes at least 50 base pairs (bp) in size. SVs are closely related to diverse human diseases Weischenfeldt et al. (2013); Lupski, (2015), such as autism Pinto et al. (2010); Sanders et al. (2012); Chen et al. (2017) and schizophrenia (Sebat et al., 2007; Stefansson et al., 2008; Walsh et al., 2008; Kirov et al., 2012). Compared with single-nucleotide variations (SNVs), SVs contain more nucleotides and are considered to be higher correlated with evolution, genetic diversity and disease-causing mutations (Stankiewicz and Lupski, 2010; Weischenfeldt et al., 2013; Abel et al., 2020).

Since the size of SV can exceed 1,000 bp, SV detection will be limited by the size of DNA fragments in sequencing. Furthermore, if SVs occur in repetitive regions with high mutation rate, it will be more difficult for detection (Hills et al., 2007; Hastings et al., 2009; Hodgkinson et al., 2012).

In view of the above problems, short-read data may have some difficulties while long-read data can be a good solution (Pollard et al., 2018; Liu et al., 2019). In recent years, long-read technologies have been developed rapidly Amarasinghe et al. (2020) and used in the discovery of SVs with complex forms (Aneichyk et al., 2018; Song et al., 2018; Ishiura et al., 2019; Zeng et al., 2019; Logsdon et al., 2020). The size of DNA fragment sequenced by long-read technologies is usually larger than 1,000 bp, which can cover the range of large SV and contain much context information (Chaisson et al., 2015). It ensures the advantages of long-read technologies in SV detection, especially in the complex repetitive regions of the genome. Characterized by high incidence rate of SVs and high complexity, repetitive regions are an important and challenging problem in SV detection (Sudmant et al., 2015; Zook et al., 2020). However, the performance of SV detection pipelines based on long-read data applied in repetitive regions still need to be analyzed.

Therefore, in this study, we selected three commonly used long-read-based pipelines Kosugi et al. (2019); Logsdon et al. (2020), namely PBSV Wenger et al. (2019), Sniffles Sedlazeck et al. (2018) and PBHoney English et al. (2014), and comprehensively evaluated their performance on SV detection. Using the benchmark established by the Genome in a Bottle (GIAB) Consortium Zook et al. (2020) as the gold standard, we evaluated the precision, recall and F1 score of these pipelines. The comparison included the comparison between insertions and deletions, the comparison among four size ranges of SVs and the comparison between SVs in TRRs and SVs outside TRRs. The F1 scores of Sniffles were 0.60 in TRRs and 0.76 outside TRRs. Similarly, The F1 scores of PBSV were 0.59 and 0.74 in and outside TRRs, respectively. The performances of the two pipelines were generally higher than that of PBHoney. For the three pipelines, the performances in TRRs were lower than those outside TRRs, which indicated that SV detection in TRRs was more difficult than that outside TRRs. Concerning the type of SVs, it was found that large insertions (> 1,000 bp) were the most difficult to detect while large deletions were easy to precisely detect, especially in TRRs. In addition, we also analyzed the potential performance of three pipelines on detecting *de novo* SVs. The results suggested that long-read technologies and the SV detection pipelines still need further development for the precise detection of *de novo* SVs.

## Materials and Methods

### Datasets

The long-read sequencing data of an Ashkenazim Jewish trio Zook et al. (2016) were used in our study. Subreads datasets of the son (HG002), the father (HG003) and the mother (HG004) were downloaded from GIAB (https://ftp-trace.ncbi.nlm.nih.gov/giab/ftp/data/AshkenazimTrio/). The average coverages of the trio are approximately 69X, 32X and 30X, and their N50 subread lengths are 11,087, 10,728, and 10,629 bp.

### Benchmark

The benchmark is established by GIAB for HG002 on GRCh37, which was downloaded from GIAB FTP site (https://ftp-trace.ncbi.nlm.nih.gov/ReferenceSamples/giab/data/AshkenazimTrio/analysis/NIST_SVs_Integration_v0.6/). The benchmark dataset contains close to 100% of true insertions and deletions in the specific regions. According to the guidance of the benchmark, we used the SVs with the FILTER field “PASS” in the Tier 1 vcf, including 12,745 isolated, sequence-resolved insertion (7,281) and deletion (5,464) calls. The benchmark regions include 34,830 large regions, of which 15% are within 1,000–10,000 bp and 82% are over 10,000 bp. Through the manual inspection in the benchmark work, it was found that approximately 5% of true insertions in the benchmark regions might be missing. Therefore, when comparing callsets (especially from long-read data) with the benchmark, it is possible to misjudge some true insertions. When making the comparison, we first selected the SVs in the benchmark regions, and then compared these SVs with the benchmark SVs.

### Structural Variation Detection Pipelines

In this study, we used three long-read-based pipelines named PBSV (version 2.2.2; https://github.com/PacificBiosciences/pbsv), Sniffles (version 1.0.11; https://github.com/fritzsedlazeck/Sniffles) and PBHoney (in PBSuite-15.8.24; http://sourceforge.net/projects/pb-jelly/). For PBSV and Sniffles, subreads were aligned to reference genomes GRCh37 by PBMM2 and NGMLR, respectively. After the help of SAMtools, SVs were called by PBSV and Sniffles. PBHoney includes two parts of results, namely Tails (based on interrupted mapping) and Spots (based on intra-read discordance). There were too few results in the Tails part to compare with other pipelines, thus the result of the Tails part was separately shown in Supplementary Figure S1. Because of the complexity of parameter optimization in Spots and the time-consume of recommended aligner BLASR, following a previous work Kosugi et al. (2019), we used NGMLR to align the subreads and detected SVs with custom-made parameters for insertions and deletions. SVs with < 0.2 of the value, which was calculated by dividing the szCount tag with the coverage tag, were filtered out.

In these callsets, we only summarized the variations ≥ 50 bp. SVs with the type “BND” (breakpoint end) were excluded. In this study, we only focused on the SVs on the autosomes and sex chromosomes.

### The Metrics for Comparison

During comparison, we mainly considered the type consistency, the distance between breakpoints and the proportion of the reciprocal overlap. For compared insertions, if the distance of breakpoints was within 200 bp, they were considered the same. For compared deletions, the called SV needed to exhibit ≥ 50% reciprocal overlap with the reference SV. When comparing the callset with regions (i.e. the benchmark regions and tandem repeat regions), it was only required that breakpoints overlapped with these regions. When comparing the overlap among pipelines, the callset with more SVs was chosen as the comparison benchmark. The code used for comparison are available at GitHub (https://github.com/cic-gmk/DNSV).

When comparing the callsets with the benchmark, the precision, recall and F1 score were calculated *via* the following equations:

$$\text{Precision }=\;\frac{\text{TP}}{\text{TP}+\text{FP}}$$

$$\text{Recall }=\;\frac{\text{TP}}{\text{TP}+\text{FN}}$$

$$\text{F}1\text{ score }=\;2\times \frac{\text{Precision}\times \text{Recall}}{\text{Precision}+\text{Recall}}$$

where TP, FP and FN are the numbers of true positives, false positives and false negatives. TP + FP is equal to the number of the called SVs. TP + FN is equal to the number of the benchmark SVs.

### Tandem Repeat Regions

The repeats used in our study were annotated in the annotation file of hg19, which can be obtained at the download site of UCSC Genome Browser (Fernandes et al., 2020; Navarro Gonzalez et al., 2020) (http://hgdownload.soe.ucsc.edu/goldenPath/hg19/database/rmsk.txt.gz). “Simple repeats” and “Satellites” were selected as the TRRs from the file. “Simple repeats” are short pattern tandem repeats and “Satellites” are medium to long pattern tandem repeats. SVs were divided into two parts according to whether they were in TRRs or not.

## Results

### The Landscape of Structural Variation Callsets

**FIGURE 1:**
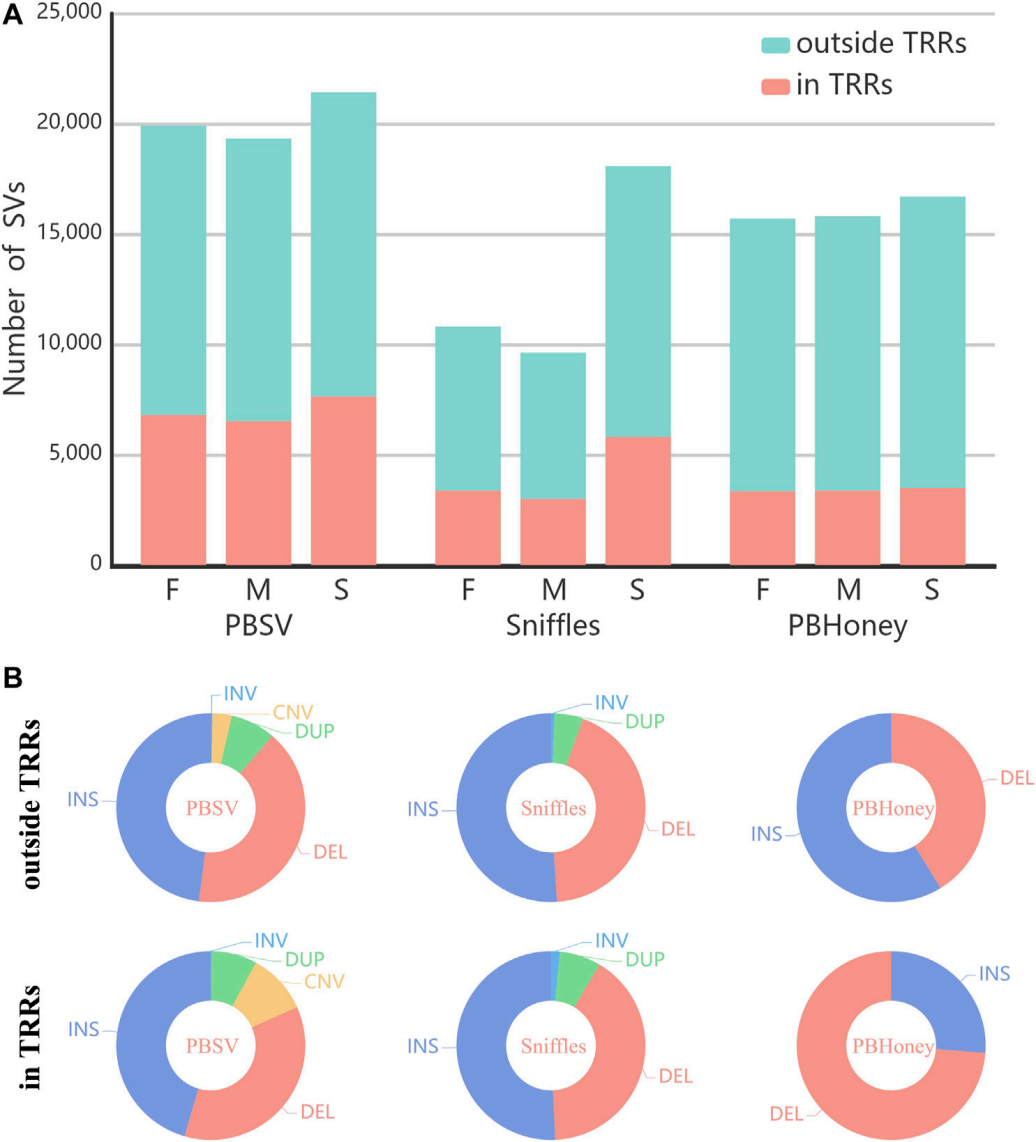
**(A)** The number of SVs in the callsets of the parents and the son (F, Father; M, Mother; S, Son) detected by PBSV, Sniffles and PBHoney. **(B)** The type distribution of the whole callsets of the trio detected by three pipelines in/outside TRRs.

The numbers of SVs detected by the three pipelines are shown in Figure 1A. Among the three pipelines, PBSV detected the largest number of SVs and Sniffles detected the least number of SVs. For all the pipelines, the numbers of detected SVs of the son were more than those of the parents mainly due to the higher coverage of the sequencing data of the son. For Sniffles, largest difference in the numbers of detected SVs existed between the son and the parents. Because of the high mutation rate of TRRs Hills et al. (2007); Hastings et al. (2009), although the abundance of TRRs accounts for only about 10% of the human genome Benson, (1999), a number of SVs were still detected in TRRs in the callsets of the trio by three pipelines (PBSV 35%, Sniffles 32%, PBHoney 21%). Figure 1B shows the type distribution of all SVs detected by each of the three pipelines. Although insertions are more difficult to detect Zook et al. (2020), the proportions of insertions detected by three pipelines were higher than those of deletions, except for PBHoney in TRRs. The size distribution of SVs detected by each of the three pipelines is provided in Supplementary Figure S2. It was found that the number of detected SVs decreased fast as the size of SVs increased. Insertions were generally in the majority when the size < 1,000 bp, but the proportion of deletions increased with the increase of size. In addition, we also investigated the distribution of the percentage of SVs across chromosomes for pipelines (Supplementary Figure S3).

**FIGURE 2:**
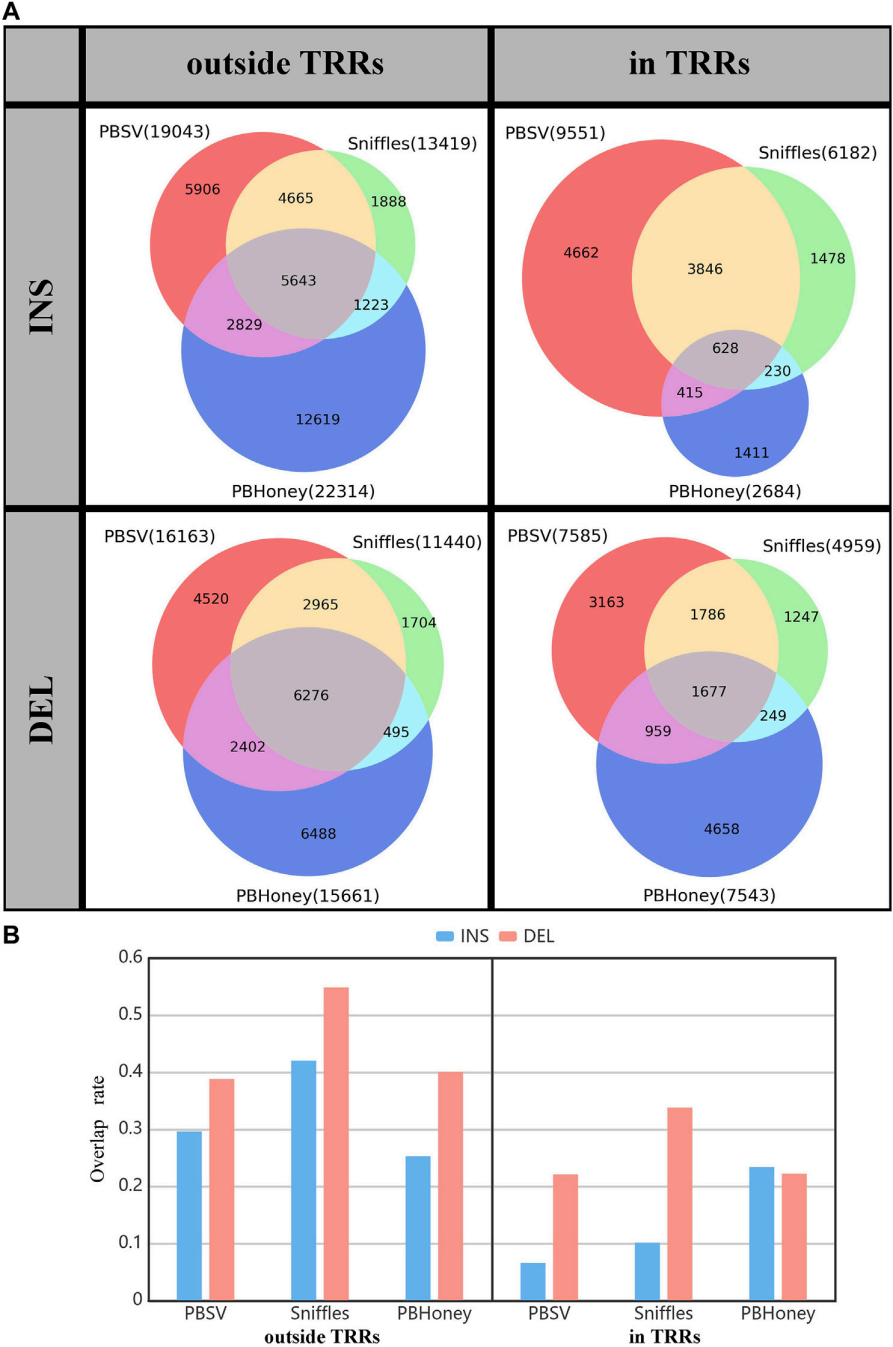
**(A)** The overlap among callsets detected by three pipelines divided by the type and the location relation with TRRs of SVs. **(B)** The overlap rate of three pipelines. The gray part in Figure 2A shows the overlap SVs.

We summed the overlap among the callsets of each person detected by three pipelines for comparison (Figure 2). For the SVs outside TRRs, the overlap proportion of SVs detected by Sniffles was the highest, and close to 42% (5,643/13,419) of insertions and 55% (6,267/11,440) of deletions can be detected by the other two pipelines. For SVs in TRRs, when comparing Sniffles with PBSV, about 72% ((3,846 + 628)/6,182) of insertions and 70% ((1,786 + 1,677)/4,959) of deletions identified by Sniffles can be detected by PBSV. However, only 628 insertions detected by PBHoney in TRRs were involved in the callsets of PBSV and Sniffles due to the insufficient ability of PBHoney for detecting insertion in TRRs. It can be seen from Figure 2B that, except for PBHoney in TRRs, the overlap rates of insertions were lower than those of deletions. For the three pipelines, the overlap rates in TRRs were lower than those outside TRRs, suggesting that the difference among the callsets from different pipelines in TRRs was large.

### Evaluation on the Performance of Pipelines

The benchmark used in our study defines the comparing regions, in which the benchmark contains close to 100% of true insertions and deletions. Therefore, we compared the callsets detected by three pipelines with the benchmark callset in the comparing regions.

**FIGURE 3:**
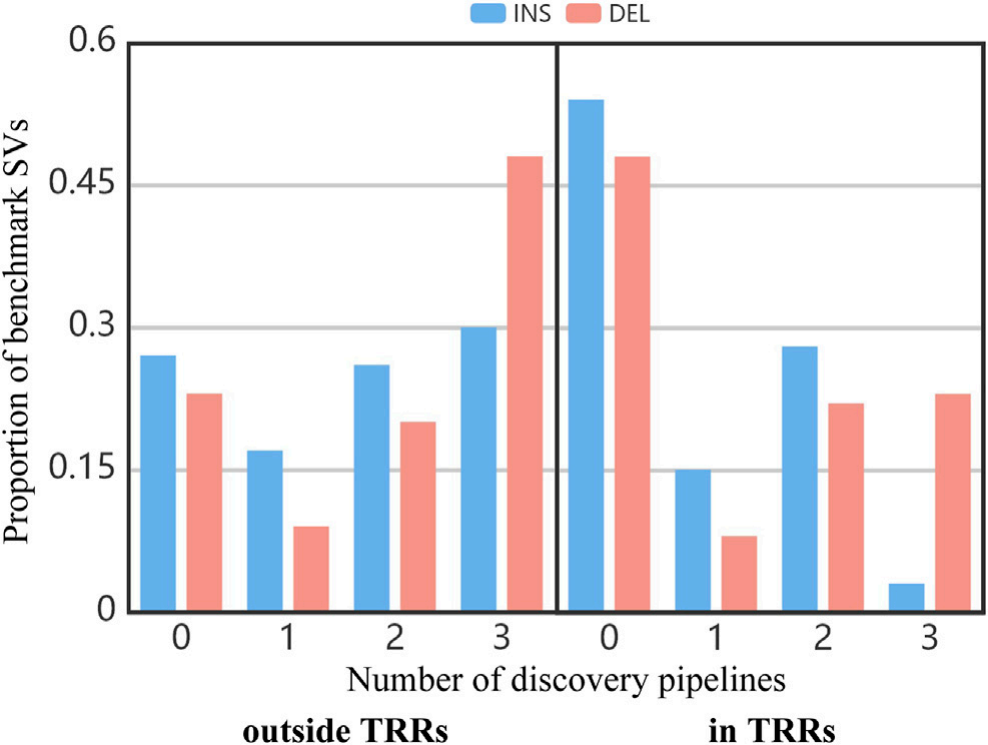
The proportion of benchmark SVs concurrently detected by different number of pipelines.

Figure 3 shows the proportion of benchmark SVs concurrently detected by different number of pipelines. In the whole benchmark callset, close to 25% of benchmark SVs outside TRRs and 51% of benchmark SVs in TRRs cannot be discovered by any pipeline (the bar of “0”). It suggested that SVs in TRRs were more difficult to detect. Similarly, the proportion of benchmark SVs concurrently detected by three pipelines in TRRs was obviously lower than that outside TRRs (the bar of “3”), which agreed with the overlap results among three pipelines (Figure 2).

**FIGURE 4:**
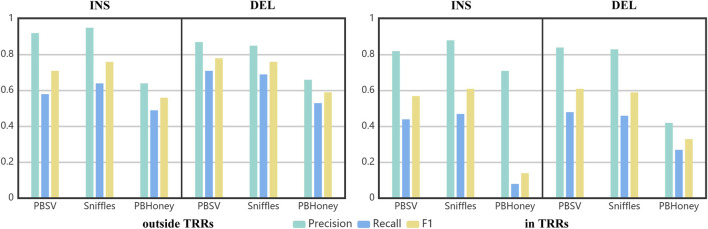
The precision, recall and F1 of three pipelines in detecting insertions and deletions in/outside TRRs.

Using the benchmark as the golden standard, the precision, recall and F1 score of three pipelines are shown in Figure 4. The precisions achieved by PBSV and Sniffles were higher than 80% both in and outside TRRs, indicating that the SVs detected by these two pipelines were relatively precise. The precision of PBHoney was the lowest, suggesting that more false positives existed in the callset of PBHoney. For all the pipelines, the recalls were under 80 and 50% outside and in TRRs, respectively. It suggested that there were still a number of SVs omitted by the three pipelines. The recall of insertions detected by PBHoney in TRRs was especially low (8%), suggesting that its detection ability of insertions in TRRs was suboptimal. For all the three pipelines, the F1 scores in TRRs were obviously lower than those outside TRRs, indicating the detection of SVs in TRRs was more challenging. In addition, because the son’s SVs are inherited from the parents, we also made comparison between the callsets of the parents and the benchmark (Supplementary Figure S4). The nominal precisions of the callsets of the parents were clearly lower than those of the son mainly because the benchmark was constructed only based on the sequencing data of the son. It suggested that the benchmark construction in future need to consider the diversity of the population.

**FIGURE 5:**
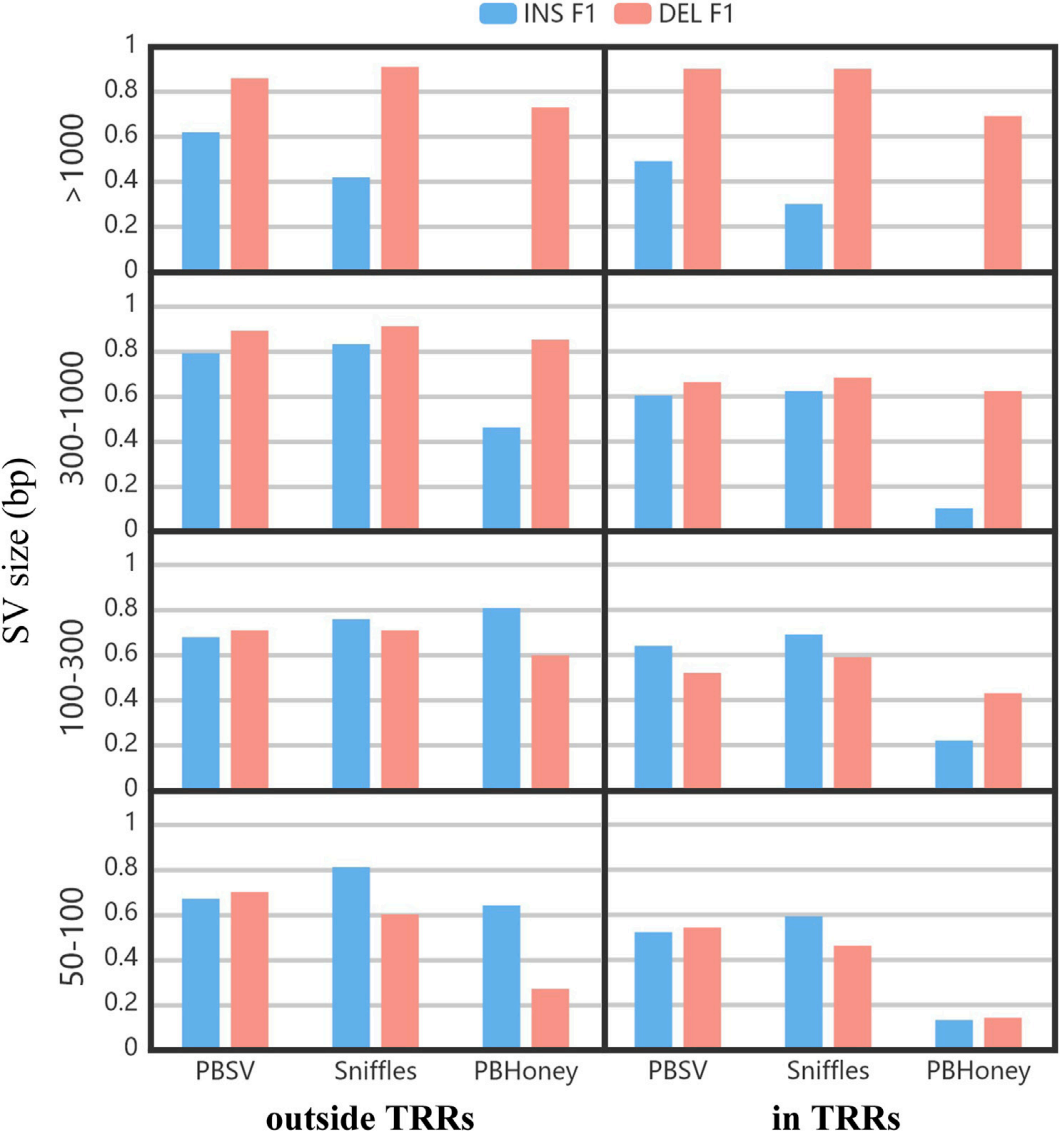
The F1 of insertions and deletions divided by the size and the location relation with TRRs of SVs. The size ranges included 50–100 bp, 100–300 bp, 300–1,000 bp and > 1,000 bp.

Figure 5 shows the impact of the size of SVs on the detection ability of three pipeline. The F1 scores of PBSV and Sniffles were relatively stable with the increase of the size of SVs. However, the size of SVs induced a clear impact on PBHoney. Especially when the size of SVs was more than 1,000 bp, PBHoney can hardly detect true insertions. For all the three pipelines, the F1 scores of large insertion detection (>1,000 bp) were obviously lower than those of large deletion detection, suggesting that the detection for large insertions were more challenging.

### Potential Performance of Three Pipelines on Detecting *de novo* Structural Variations

**FIGURE 6:**
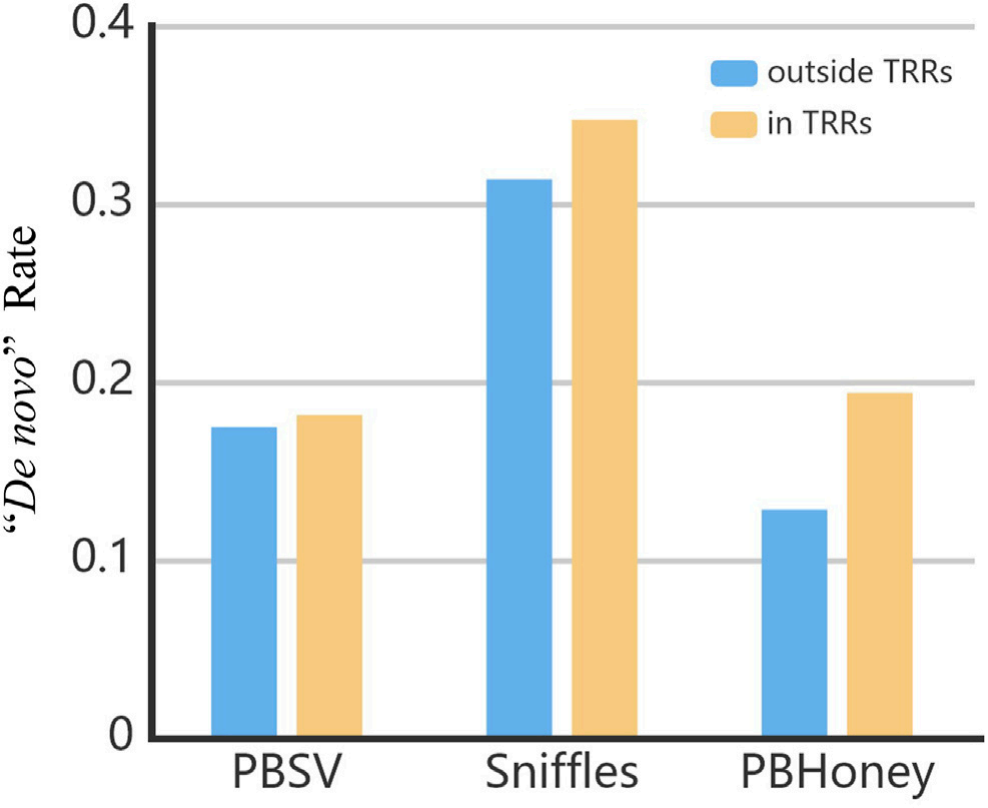
The “*de novo*” rate in the callsets of three pipelines in and outside TRRs.

The mutations that only occurred in the child rather than the parents are generally called *de novo* mutations (Conrad et al., 2011; Veltman and Brunner, 2012). We calculated the rate of “*de novo*” SVs by dividing the number of the SVs only detected from the son by the number of all SVs detected from the son. As shown in Figure 6, the “*de novo*” rate of Sniffles was 32%, which was higher than that of PBSV (18%) and PBHoney (14%). These rates were much higher than the actual *de novo* rate (Veltman and Brunner, 2012). It indicated that a large number of false positive *de novo* SVs existed in the callsets, which may also be attributed to Mendelian inheritance errors Pilipenko et al. (2014); Kothiyal et al. (2019), the false positive SVs of the son and the false negative SVs of the parents. Our results suggested that long-read technologies and the detection pipelines still need further improvement for detecting *de novo* SVs when applying to the exploration of mechanisms of rare diseases. It is a considerable challenge to reduce the false positives and false negatives in SV detection in future study.

## Discussion

The relatively large size of SVs and the complex repetitive context make SV detection challenging. Because long-read sequencing data contain abundant context information, it can perform well in SV detection. Therefore, we comprehensively analyzed the performance of three commonly used long-read SV detection pipelines. Our results showed that the overlap proportion among the callsets of the pipelines in TRRs was generally lower than that outside TRRs. Comparing callsets with the benchmark, the precisions, recalls and F1 scores of these pipelines in TRRs were obviously lower than those outside TRRs. These results suggested that the detection of SVs in TRRs was more difficult than that outside TRRs.

As shown in Figure 4, the F1 scores of PBSV and Sniffles were similar, and higher than that of PBHoney. With the default recommended parameter, preferable results can be obtained by PBSV and Sniffles. As shown in Figure 5, the F1 scores of PBSV and Sniffles did not change a lot with the increase of the size of SVs except for the detection of insertions larger than 1,000 bp. But the detection of both insertions and deletions with PBHoney was clearly influenced by the size of SVs. In fact, as shown in Supplementary Figure S2, it was difficult for PBHoney to detect SVs above 4,000 bp. For PBHoney, it was necessary to make proper settings and filter process for SVs with different types. In addition, there were 3% of the son’s callset of PBSV and 45% of the son’s callset of Sniffles marked with the label “IMPRECISE”, which indicated the probably insufficient precision of SVs. These SVs were mainly composed of insertions (95% for PBSV and 71% for Sniffles). Interestingly, the precision of SVs tagged “IMPRECISE” in PBSV was really low (15%), but for Sniffles, the precision was still high (81%), which meant there was no need to filter these SVs in Sniffles.

In our study, we selected SVs (≥ 50 bp) from the benchmark for comparison. If more variations was selected, such as using the cutoff of variations ≥ 30 bp, it would lead to higher precision and lower recall (Kosugi et al., 2019). Our results showed that, even with high precision, no pipeline can achieve very high recall in SV detection. Therefore, it is necessary to integrate different pipelines for generating a comprehensive callset. Integrating Sniffles and PBHoney, NextSV Fang et al. (2018) had been developed to detect SVs from low-coverage long-read sequencing data and achieved better performance than a single pipeline. In addition, a pipeline with multiple algorithms can be developed to optimize and simplify the procession of SV detection.

Previous studies have found that long-read sequencing can identify pathogenic SVs of rare genetic diseases which cannot be identified by short-read sequencing, such as the pathogenic SVs of Carney complex Merker et al. (2018) and progressive myoclonic epilepsy (Mizuguchi et al., 2019). In the study of Carney complex, the pathogenic SV was identified by pipeline detection followed by manual screening and analysis. Since the SV was not detected from the parents, the pathogenic SV was also proved to be a *de novo* SV. However, it was difficult to identify pathogenic SVs from “*de novo*” SVs using long-read sequencing. It is known that the number of *de novo* mutations in heredity is very small (Conrad et al., 2011; Veltman and Brunner, 2012). But compared with the number of *de novo* single-nucleotide variations detected from short-read data Liang et al. (2019), the number of “*de novo*” SVs detected from long-read data was too large. And the precision was too high when comparing the “*de novo*” SVs with the benchmark (Supplementary Figure S5). Therefore, these SVs cannot be simply regarded as true *de novo* SVs. In order to analyze true *de novo* SVs, more true SVs need to be detected from parents. As shown in Figure 1A, in each pipeline, the numbers of SVs of the parents were obviously smaller than that of the son due to the lower coverages of the parents. It suggested that the sequencing coverages of the trio need to be ensured. Also, for high precision and recall of the detection of *de novo* SVs, the precision and recall of SV detection pipelines still need to be improved.

## Conclusion

In this study, we thoroughly compared three commonly used SV detection pipelines and found that the precisions of PBSV and Sniffles were generally similar, and higher than PBHoney. The recalls of the three pipelines were still suboptimal. The performances of PBSV and Sniffles were relatively stable with the increase of the size of SVs, while the performance of PBHoney varied largely. The performances of the three pipelines in TRRs were obviously lower than those outside TRRs, indicating that SV detection in TRRs was more difficult. Comparing insertions with deletions, the detection of large insertions was obviously more difficult than that of large deletions. Our findings can be helpful for conducting the SV detection in the mechanism exploration of rare diseases.

## Data Availability

The original contributions presented in the study are included in the article/Supplementary Material, further inquiries can be directed to the corresponding author.
