# Supplementary material for: Comparative Analysis for the Performance of Long-Read-Based Structural Variation Detection Pipelines in Tandem Repeat Regions
Source: Front Pharmacol. 2021 Jun 7;12:658072. doi: 10.3389/fphar.2021.658072 (PMC8215501; doi:10.3389/fphar.2021.658072)
Supplement: Supplementary file 1 [file DataSheet1.docx]

Supplementary Material

# Supplementary Figures

**
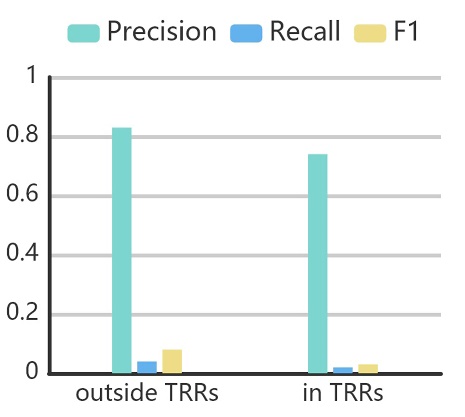
**

**Supplementary Figure S1.** The precision, recall and F1 score of PBHoney-Tails in and outside TRRs.

**
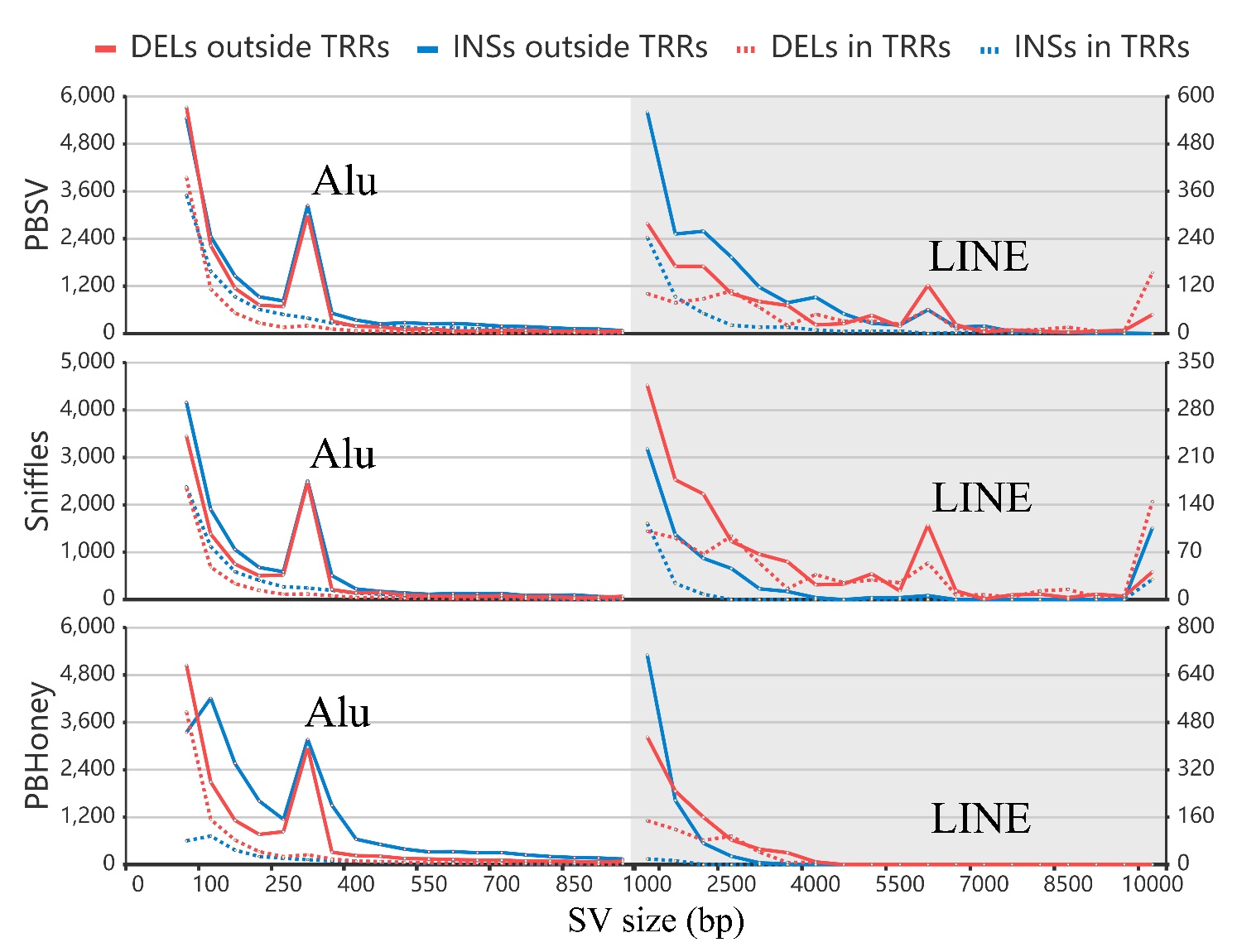
**

**Supplementary Figure S2.** The size distribution of insertions and deletions. The blue lines were insertions while the red lines were deletions. The dotted line meant in TRRs while the solid line meant outside TRRs. Before 1000 bp, the interval was 50 bp. After 1000 bp, the interval was 500 bp. The expected Alu peak was near 300 bp and the LINE peak was near 6000 bp.

**
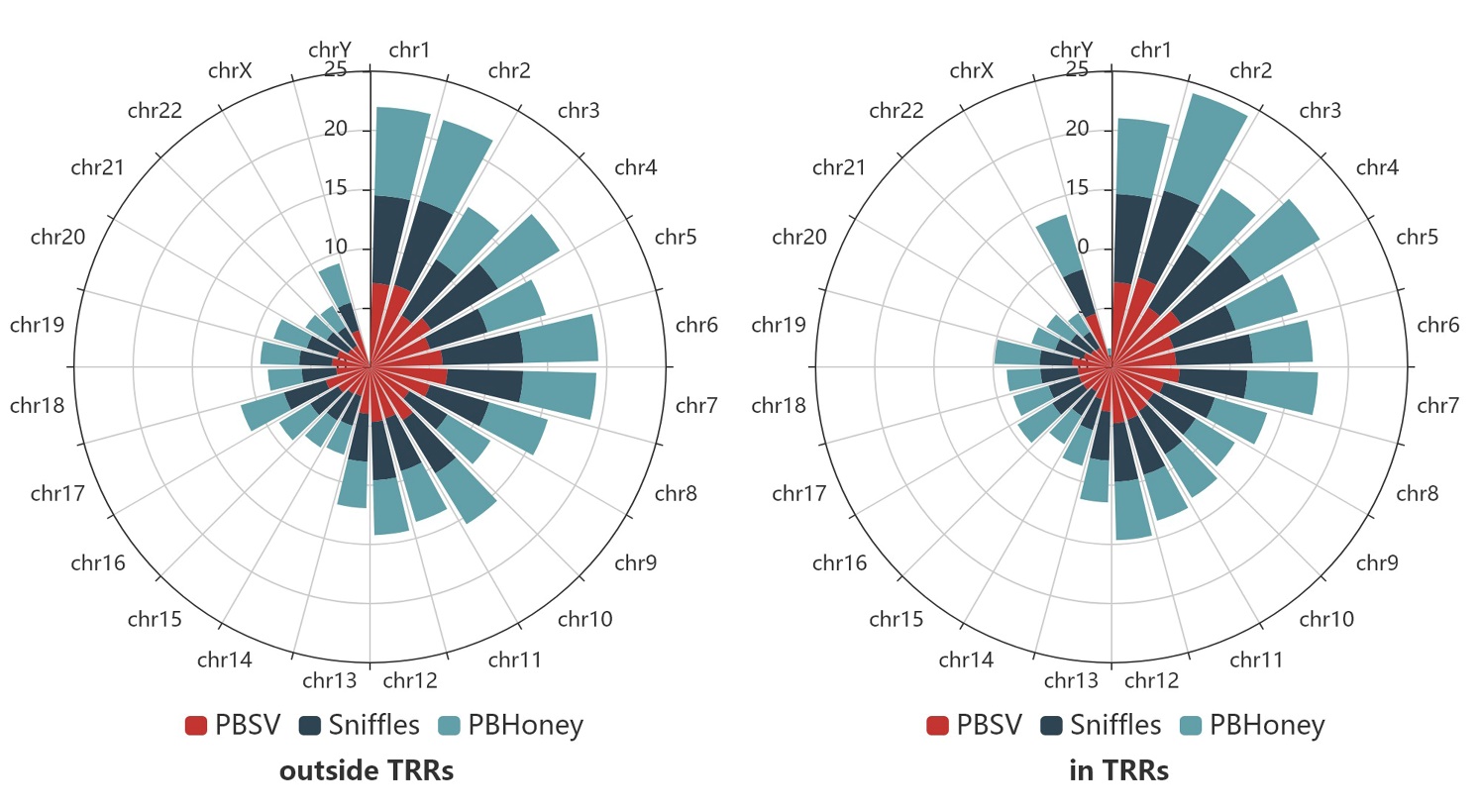
**

**Supplementary Figure S3.** The distribution of the percentage of SVs across chromosomes for pipelines in/outside TRRs.

**
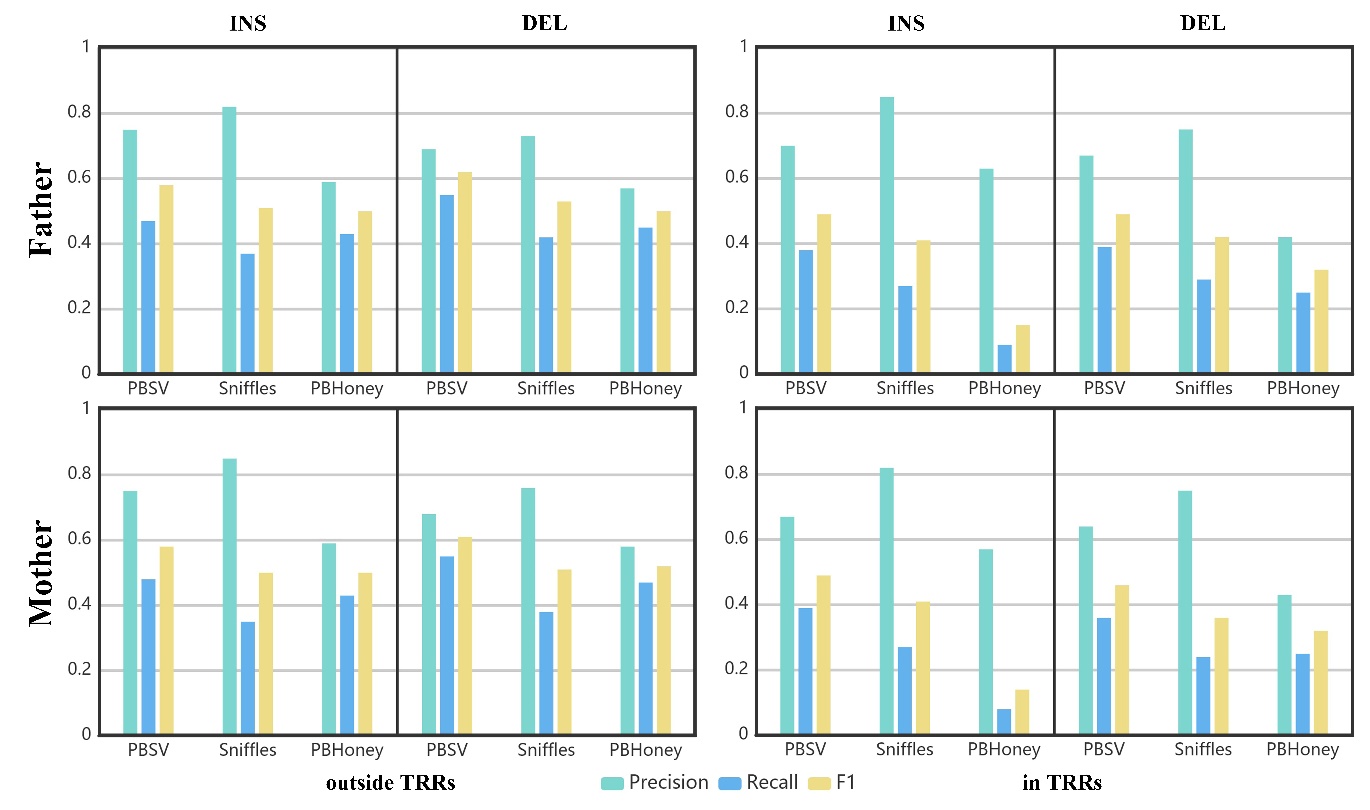
**

**Supplementary Figure S4.** The comparison between the callset of the father or the mother and the benchmark.

**
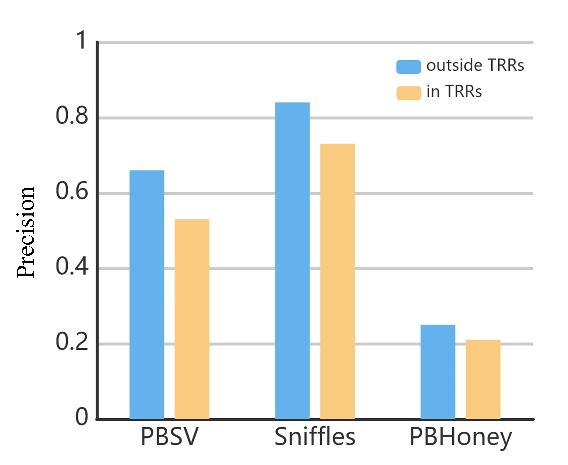
**

**Supplementary Figure S5.** The precision of “*de novo* SVs” in and outside TRRs. The true positives did not mean the true positives *de novo* SVs. They could also be the false negative of the callsets of the parents.
